# Effects of Bi–Sn–Pb Alloy and Ball-Milling Duration on the Reactivity of Magnesium–Aluminum Waste-Based Materials for Hydrogen Production

**DOI:** 10.3390/ma16134745

**Published:** 2023-06-30

**Authors:** Olesya A. Buryakovskaya, Grayr N. Ambaryan, Alexey B. Tarasenko, Musi Zh. Suleimanov, Mikhail S. Vlaskin

**Affiliations:** Joint Institute for High Temperatures of the Russian Academy of Sciences, 125412 Moscow, Russia; ambaryan1991@gmail.com (G.N.A.);

**Keywords:** magnesium scrap, ball milling, hydroreactive materials, low melting point alloy, microstructural transformation, intermetallides, aqueous salt solutions, hydrogen generation

## Abstract

In the present study, composite materials were elaborated of mixed scrap of Mg-based casting alloys and low melting point Bi–Sn–Pb alloy by high energy ball milling, and their reactivity in NaCl solution with hydrogen release was tested. The impacts of the additive content and ball milling duration on their microstructure and hydrogen generation performance were investigated. Scanning electron microscopy (SEM) analysis revealed significant microstructural transformations of the particles during milling, and X-ray diffraction analysis (XRD) proved the formation of new intermetallic phases Mg_3_Bi_2_, Mg_2_Sn, and Mg_2_Pb. The said intermetallic phases were anticipated to act as ‘microcathodes’ enhancing galvanic corrosion of the base metal. The dependency of the samples’ reactivity on the additive content and milling duration was determined to be nonmonotonic. For the samples with 0, 2.5, and 5 wt.% Rose alloy, ball-milling during 1 h provided the highest hydrogen generation rates and yields (as compared to 0.5 and 2 h), while in the case of the maximum 10 wt.%, the optimal time shifted to 0.5 h. The sample activated with 10 wt.% Rose alloy for 0.5 h provided the highest ‘metal-to-hydrogen’ yield and rapid reaction, thus overperforming those with lower additive contents and that without additives.

## 1. Introduction

Global over-reliance on fossil fuels creates a good deal of problems. The environmental impacts include emission of greenhouse gases [1,2] and pollution of water sources [3,4], while regional economic development may suffer from shortages and disruptions in the energy carrier’s supply [5,6]. Overcoming those complex issues requires transition to renewable clean energy sources. Hydrogen represents an environmentally friendly secondary energy carrier that can serve as temporary storage for green energy; its chemical energy can be converted into electricity and/or heat by means of fuel cells, internal combustion engines, catalytic combustors, and hydrogen-steam or hydrogen-fired gas turbines [7,8,9,10,11,12]. This gas can be derived from biomass or obtained by water splitting using solar, wind, or hydropower electricity [13,14,15,16].

To ensure evolution toward the ‘hydrogen economy’, modern infrastructure should be designed. While plenty of practical techniques for hydrogen production have been proposed, its storage and transportation still pose quite a challenge. For those applications, the best known methods currently include compression to 350–700 bar [17,18], liquification at −253 °C [19,20], and storage in a bound form using zeolites, metal–organic frameworks, covalent organic frameworks, carbon-based materials, metal hydrides, ammonia, methanol, etc. [21,22,23]. Along with all the advantages, each of the listed systems has its downsides. Thus, high-pressure systems bear the safety risks of gas leakage because of the degradation of the material’s properties (burst, fiber damage, fatigue life failures, collapse and blistering of the liner, typical for types III and IV composite hydrogen storage tanks) [24,25]. The cryogenic method requires high capital expenses and heavy energy consumption [26,27], and the rest of the methods still fail to provide both high hydrogen storage capacity and acceptable hydrogenation/dehydrogenation conditions [28,29].

An alternative to the said approaches is the implementation of Al and Mg, which react with water with hydrogen evolution. Although under normal conditions the reaction between those metals and water is hindered by their surficial oxide films or ceases soon after beginning owing to the formation of dense hydroxide layers, many useful activation techniques have been proposed [26]. Thus, implementation of nanometer-sized Mg and Al powders with extremely high specific surface areas was reported to enable their entire oxidation at room temperature [30,31]. Some approaches included disruption of the product layer by grinding or friction between particles under intensive mixing [32,33], ultrasonic treatment, and laser irradiation [34,35,36]. Hydrothermal temperatures (over 100 °C) are known to increase the diffusivity of water (liquid or gaseous) through the protective oxide layer or product deposit [37,38,39]. The original passive film and generating product layers can be removed chemically by their transformation into soluble compounds from their reactions with salt, acid, or alkali (for Al) aqueous solutions [40,41,42,43,44,45,46,47,48]. Introduction of metal additives (e.g., Ni, Co, Fe, Cu, Sn, Bi, Zn) to the basic Mg and Al materials by alloying or high energy ball milling was proved to enhance their corrosion with hydrogen production [49,50,51,52,53,54,55]. Another notable effect is embrittlement of the Al matrix based on low melting point Ga and its alloys with In, Sn, Zn, Bi, etc. [56,57,58,59,60]. One more widely used activation method is the preparation of composite powder materials by ball milling of Al and Mg with various additives, contributing to the particle size reduction and preventing their agglomeration (Al_2_O_3_, NaCl, FeCl_3_, AlCl_3_, CoCl_2_, KCl, g-C_3_N_4_, LiH, graphite, MoS_2_, MoO_2_, MoO_3_, etc.) [61,62,63,64,65,66,67,68,69,70].

Conversion of Mg and Al into hydrogen (and ecologically friendly Mg(OH)_2_ and Al(OH)_3_ or AlOOH) by their oxidation in aqueous media can be useful not only for hydrogen production but for effective waste utilization as well. For instance, metal from composite multilayer materials can be oxidized with the separation of plastic and/or paper layers [43]. Dross, scrap, and dust capable of causing uncontrollable hydrogen release upon contact with leachate or other oxidizing media can be effectively disposed of [71,72,73,74,75,76,77,78,79]. Complex alloys with high contents of alloying elements and small fractions of impurities, mixed waste compositions, and disperse materials (chips, shavings, etc.) with their extended surface partially oxidized and covered by lubricants can be recycled [80,81,82].

The mentioned mixed waste of complex alloys in a disperse form can be activated by ball milling in order to increase its reactivity for hydrogen generation. Ball milling is an advantageous technique for that purpose since it provides particle size reduction, thus increasing the specific surface area of the sample exposed to oxidizing aqueous media. It also induces the accumulation of crystal lattice imperfections favoring local corrosion attacks with hydrogen release. Another useful effect of ball milling is the distribution of an activating additive over the sample’s particles. Additives of metals ‘nobler’ than Al or Mg form microgalvanic couples with them, thus intensifying their ‘anodic’ corrosion with hydrogen evolution. The efficiency of ball milling is considerably affected by many factors. Thus, relatively small stainless steel balls (6 mm in diameter) demonstrated poor usability for particle size reduction as compared to the larger ones (≥10 mm) [83]. The grinding jar filling fractions of 45–50 vol.% [84] and 40–60 vol.% [85] proved to be more effective in comparison with the smaller and the larger ones. Denser milling ball material, tungsten carbide, appeared to outperform stainless steel [84]. Low rotational speeds (up to 200 rpm) imposed rather a small impact on the milling process as compared to 300–700 rpm [85]. A larger ball-to-powder mass ratio provided more extensive milling and contributed to particle size reduction, together with refinement of the additive inclusions and intermixing of the components in composite powders [86,87]. Process duration also was a critical parameter affecting the structural evolution of the metal particles. Thus, for aluminum powders, long ball-milling intervals (over 7–8 h) resulted in a negligible further particle size reduction, a small increase in lattice imperfections, and insignificant changes in the dispersion of the additive (alumina nanoparticles) in the aluminum matrix [88,89].

Magnesium alloy castings are known as important components of automotive, aerospace, and other transportation industries, and their machining results in the generation of tons of scrap [90,91,92,93]. Those amounts of magnesium-based waste have the potential for effective utilization with hydrogen generation. Numerous small metal mechanical enterprises currently dispose of wastes generated in their production processes, thus imposing a negative environmental impact [94]. Instead of that, they could benefit from waste utilization with in situ hydrogen production and its conversion to provide energy for balance-of-plant needs. For better efficiency, the procedure should include waste collection and utilization at the same mechanical machining enterprise that generated it. In the present study, an approach to obtaining hydrogen from the reaction between aqueous salt solution and hydroreactive samples manufactured of mixed scrap of the ML10 and ML5 alloy grades, generally equivalent to the NZK (Mg–Nd–Zn–Zr) and AZ91D (Mg–Al–Zn) respectively, was tested. The solution was 3.5 wt.% NaCl, because it simulates ecologically friendly sea water, and it is a standard solution used in many studies on the corrosion of Mg-based alloys with hydrogen release. The hydroreactive materials were produced via high energy ball milling. The starting materials were the said mixed scrap and commercial low melting point Bi–Sn–Pb (Rose) alloy serving as an activating additive. Bi, Sn, and Pb were reported to intensify hydrogen release accompanying the corrosion of Mg alloys owing to the formation of intermetallic phases Mg_3_Bi_2_, Mg_2_Sn, and Mg_2_Pb. With their respective corrosion potentials of −1463, −1498 and −1002 mV (measured in 3.5 wt.% NaCl solution), those compounds are ‘cathodic’ to the Mg phase (−1669 mV) [95,96,97]. In recent years, prices for bismuth have decreased considerably [98], while tin and lead are known as available and inexpensive materials. Despite its toxicity, lead is widely used in batteries for which special recycling/recovery programs exist; some tin compounds can impose toxic effects; however, leaching, roasting, and precipitation procedures can be used for the effective extraction of these metals [99,100,101]. Low melting point metals (such as In, Zn, Bi) appear to be effective for Mg activation because they readily react with the formation of complex ‘cathodic’ compounds during ball milling [102]. The Rose alloy components have low melting temperatures (271 °C for Bi, 327 °C for Pb, and 232 °C for Sn [103,104,105]). For Rose alloy, the temperature is still lower (94 °C), which is potentially useful for its effective distribution over the samples’ particles during ball milling. The aim of the present study is to investigate the effects of the additive concentration and ball-milling time on the hydrogen yield and evolution rate of the hydroreactive samples manufactured from the scrap of the ML5 and ML10 alloys.

## 2. Materials and Methods

The original material for the hydroreactive samples was represented by shavings and chips of the ML10 and ML5 alloy grades (National Standard ‘GOST 2856-79′ [106]), whose elemental compositions were generally similar to those for the AZ91D (Mg–Al–Zn) and NZK (Mg–Nd–Zn–Zr) alloys. The additive was represented by a commercial low melting point Rose alloy containing 18 wt.% Sn, 32 wt.% Pb, and 50 wt.% Bi (Technical Specification No 6-09-4065-88, ‘Rushim’ LLC, Moscow, Russia). The solution was prepared by dissolving chemically pure NaCl (National Standard GOST 4233-77 [107], ‘LabTech’ Ltd., Moscow, Russia) in distilled water.

The original scrap covered with lubricating oil was degreased using pure acetonitrile (Technical Specification No. 2636-092-44493179-04, ‘EKOS-1′ JSC, Moscow, Russia). The scrap–acetonitrile mixture was exposed to ultrasonic cleaning (1 h) in an ultrasonic bath sonicator (PSB-2835-05; ‘PSB-Gals’ Ltd., Moscow, Russia) and successive stirring (1 h) via a magnetic mixer (C-MAG; ‘HS 7 IKA-Werke’ GmbH & Co. KG, Staufen, Germany). After these manipulations, the used liquid was replaced with a fresh acetonitrile portion, and the procedures repeated. Finally, the cleaned scrap was separated and dried at room temperature for 24 h.

Composite samples were produced using a 50 mL milling pot filled in a glove box (G-BOX-F-290; ‘FUMATECH’ Ltd., Novosibirsk, Russia) under pure argon (99.993%, National State Standard GOST 10157-79 [108], ‘NII KM’ Ltd., Moscow, Russia), stainless steel balls, and a centrifugal ball mill (S 100; ‘Retsch’ GmbH, Haan, Germany). The parameters of the ball milling and samples are listed in Table 1.

**Figure 1:**
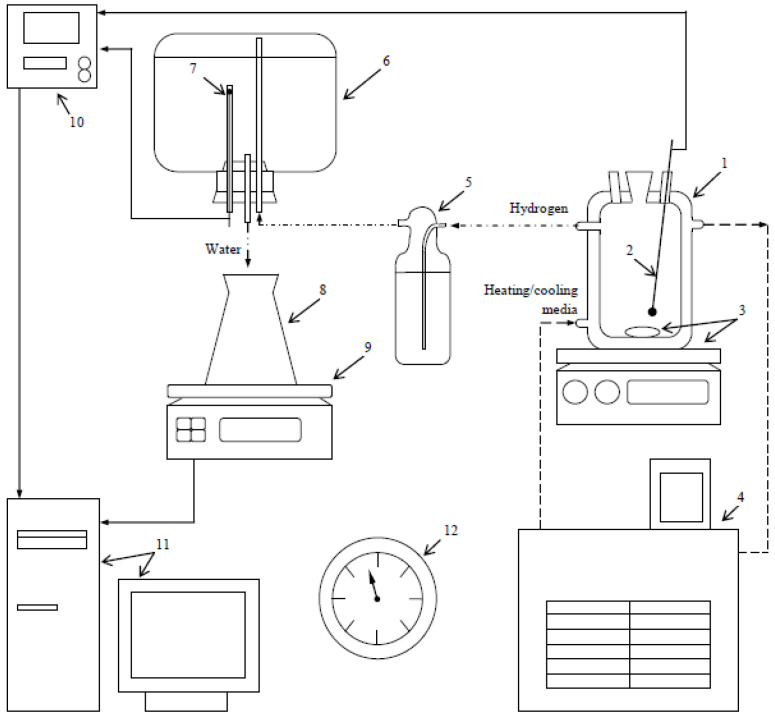
Experimental set: 1—reactor, 2—thermocouple, 3—magnetic mixer and stirring bar, 4—thermostat, 5—Drexel flask, 6—glass vessel, 7—resistance temperature detector, 8—flask, 9—scales, 10—multichannel thermometer, 11—computer, 12—barometer [83].

The experimental facility was similar to that from a preceding study [83], but with a larger glass reactor (1000 mL, JSC ‘Lenz Laborglas’, Wertheim, Germany). The reactor was equipped with a magnetic mixer (C-MAG HS 7; JSC ‘IKA-Werke’, Staufen, Germany) and a heater (CC-308B; JSC ‘ONE Peter Huber Kältemaschinenbau’, Offenburg, Germany). After pouring 1000 mL of the solution into the reactor, it was heated up; then a sample was introduced into it. The experiment parameters are given in Table 1. Hydrogen passing through a Drexel flask was collected in a glass vessel with water, which was ejected into a flask mounted onto scales (ATL-8200d1-I; ‘Acculab Sartorius Group’, New York, NY, USA). The temperatures in the reactor and glass vessel were measured correspondingly with an L-type thermocouple (TP.KhK(L)-K11; ‘Relsib’ LLC, Novosibirsk, Russia) and Pt100-type resistance temperature detector (TS-1288 F/11; ‘Elemer’ LLC, Podolsk, Russia) and displayed by the multichannel thermometer (TM 5103; ‘Elemer’ LLC, Podolsk, Russia). The temperature and mass readings were transmitted to a computer. The atmosphere pressure was measured at the beginning and the end of each experiment by a barometer (BTKSN-18; Technical Specification No. 1-099-20-85, ‘UTYOS’ JSC, Ulyanovsk, Russia) and averaged. The scheme of the experimental facility is shown in Figure 1. The hydrogen volume values at standard conditions (Standard DIN 1343: 101,325 Pa, 0 °C) were obtained using the collected data and the ideal gas law. For each of the samples, the experiment was repeated for three times. The kinetic curves and standard deviations were derived from averaging the data.

For the original materials, ball-milled samples, and solid reaction products, X-ray diffraction (XRD) analysis was conducted with a ‘Difraey 401′ diffractometer (‘Scientific Instruments’ JSC, Saint Petersburg, Russia) with Cr-Kα radiation (0.22909 nm). The XRD peaks were analyzed using an ICDD (International Centre for Diffraction Data) database (Powder Diffraction File™). The microstructure of the samples was investigated by the scanning electron microscopy (SEM) method in secondary electron (SE) and backscattered electron (BSE) imaging modes. The elemental compositions were estimated by the energy-dispersive X-ray spectroscopy (EDX) method. SEM-EDX analysis was performed by a scanning electron microscope TESCAN VEGA3 (‘Oxford Instruments’ PLC, Abingdon, UK) for all samples, excepting those without additives, whose microphotographs were taken previously using a scanning electron microscope NOVA NanoSem 650 (FEI Co., Hillsboro, OR, USA).

## 3. Results and Discussion

### 3.1. Properties of the Starting Materials and Ball-Milled Samples

As demonstrated in the previous studies [67,109], prolonged ball milling (2–4 h) combined with a relatively high 20 wt.% of low melting point Wood’s alloy diminished the hydrogen production performance for the samples produced of the same metal scrap. The measured specific surface areas of the original Mg scrap and samples ball milled for 0.5, 1, 2, and 4 h were respectively 2.567, 2.036, 2.022, <0.1, and 0.943 m^2^/g [109]. This nonmonotonic trend was caused by the microstructural evolution of the samples’ particles affected by competing agglomeration and disintegration processes driven by impacts from steel milling balls. After ‘short-time’ activations during 0.5 and 1 h, the particles looked like flattened ‘flakes’ and had average sizes (maximum Feret diameters) of 277 and 155 μm, respectively. Those milled for 2 and 4 h took the form of equiaxial solid objects with irregular shapes, 68 μm vs. 36 μm in average size. Considering the previous results, in the present study the maximum milling duration and additive content were correspondingly 2 h and 10 wt.%.

#### 3.1.1. Investigation of Microstructure and Elemental Composition

**Figure 2:**
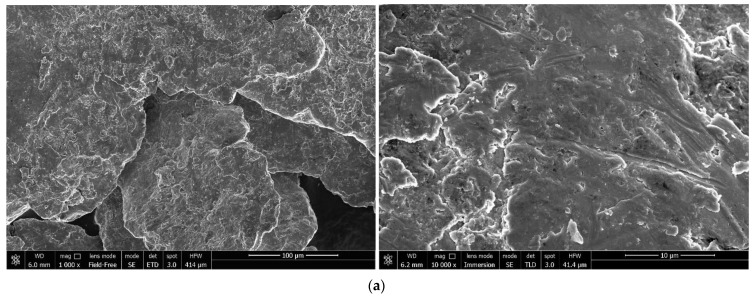

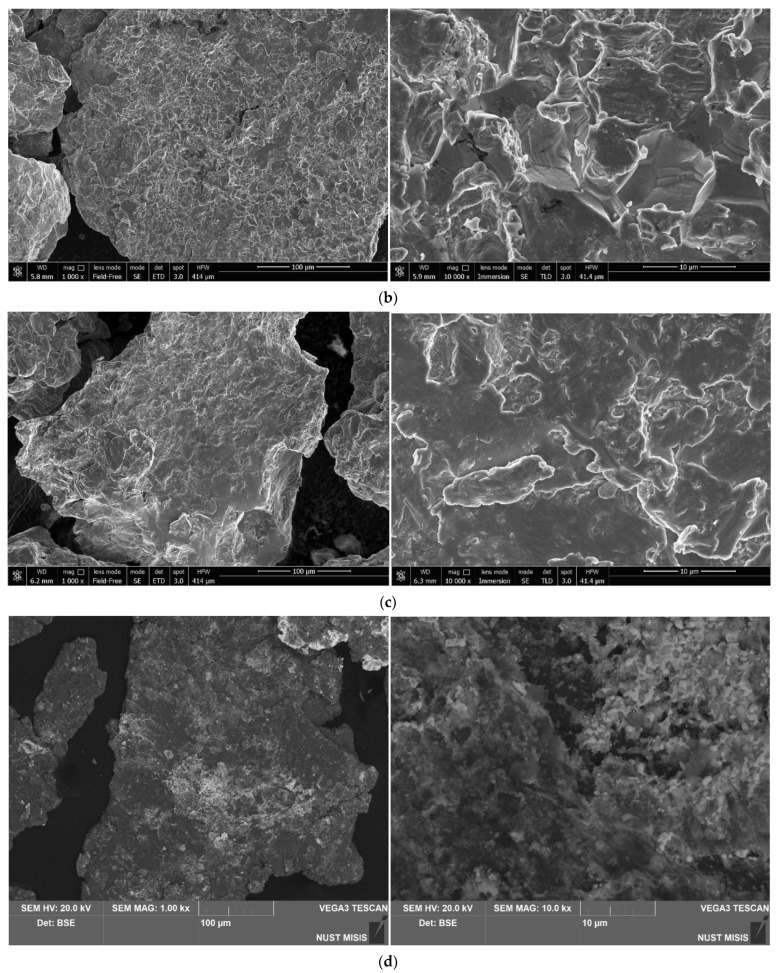

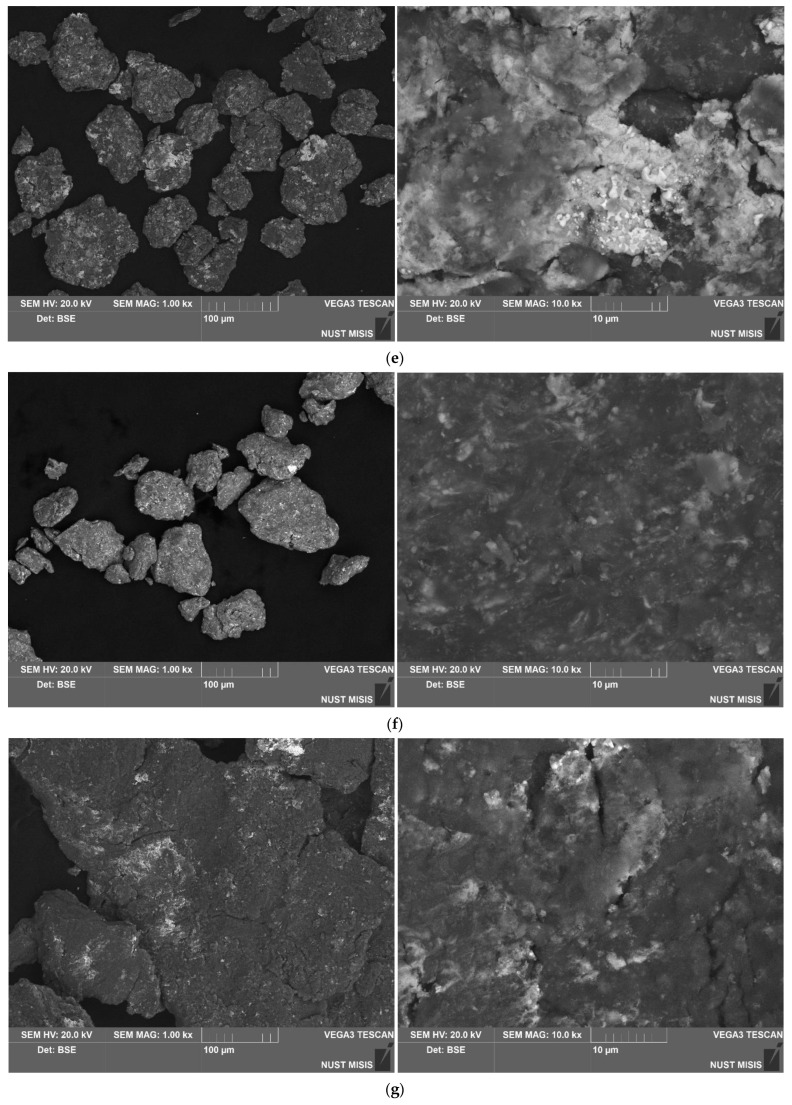

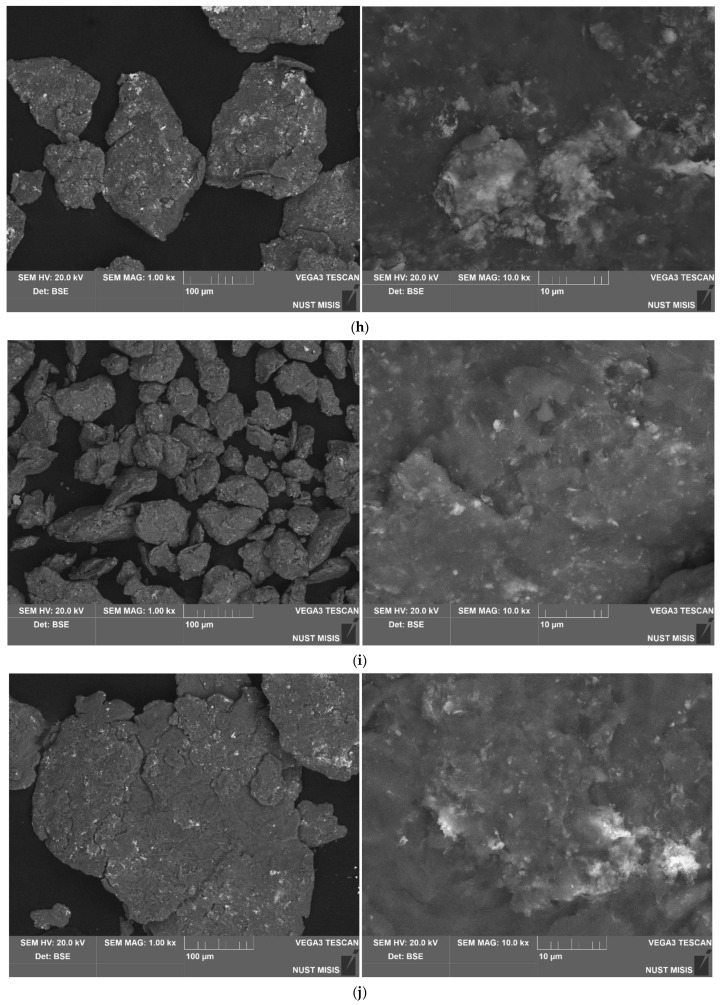

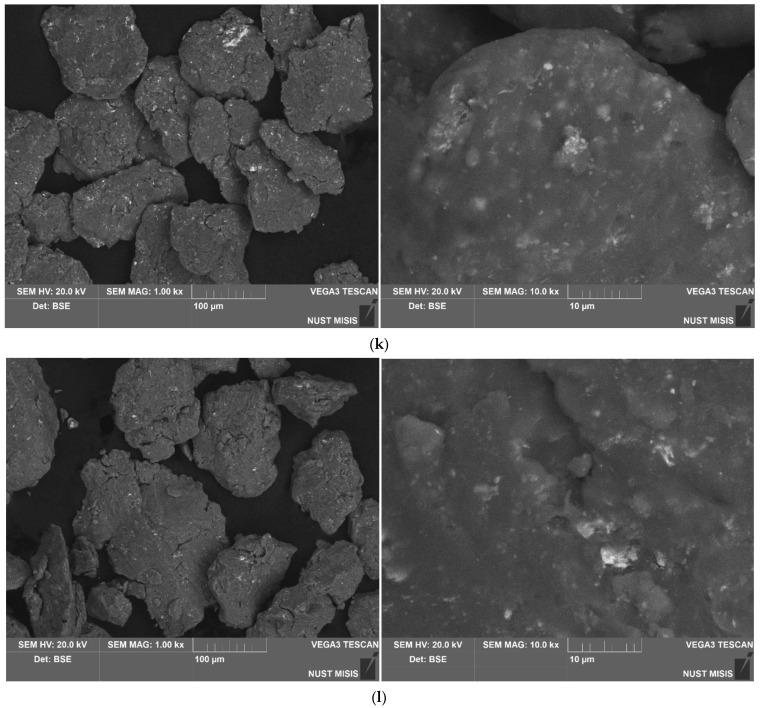
Microphotographs of the ball-milled samples: (**a**) 0.5 h milled sample without additives (SE); (**b**) 1 h milled sample without additives (SE); (**c**) 2 h milled sample without additives (SE); (**d**) 0.5 h milled sample with 10 wt.% Rose alloy (BSE); (**e**) 1 h milled sample with 10 wt.% Rose alloy (BSE); (**f**) 2 h milled sample with 10 wt.% Rose alloy (BSE); (**g**) 0.5 h milled sample with 5 wt.% Rose alloy (BSE); (**h**) 1 h milled sample with 5 wt.% Rose alloy (BSE); (**i**) 2 h milled sample with 10 wt.% Rose alloy (BSE); (**j**) 0.5 h milled sample with 2.5 wt.% Rose alloy (BSE); (**k**) 1 h milled sample with 2.5 wt.% Rose alloy (BSE); (**l**) 2 h milled sample with 2.5 wt.% Rose alloy (BSE).

Microstructures of the samples ball milled for 0.5, 1, and 2 h without additives and with different amounts of Rose alloy (10, 5, and 2.5 wt.%) are illustrated in Figure 2. The samples milled without additives were captured in the SE mode to demonstrate the surface morphology of the particles. As defined above, after 0.5 and 1 h of activation, the particles resembled flat-shaped ‘flakes’ with the traces from steel ball impacts on the surface. Under a higher magnification (see Figure 2a), voids, irregularities, and granulations roughly ~0.1 μm in size were observed. For the 1 h milled sample nearly a quarter of the particles had texturized (oriented) structures on their surface (see Figure 2b). These structures presumably resulted from polygonization and recrystallization processes known to be induced by plastic deformations of metals [110,111]. In the case of ‘prolonged’ (2 h) activation, the particles turned into compacted agglomerates of smaller particles, which resulted from fracturing of the mentioned flat-shaped ‘flakes’. As seen from the microphotographs, the described ‘reshaping trend’ was generally the same for all samples regardless of the presence or absence of the activating Rose alloy. For all samples, fractures, deformations, and small particles cold welded to the surface of the larger ones were observed. No considerable differences in the particle sizes were visually registered for the samples with the additive and without it.

Observations on the ball milling of ductile metals (such as aluminum and magnesium) revealed that short ball-milling durations led to the particles’ deformations. The particles trapped between colliding milling balls were subjected to compression and shearing loads, which induced their flattening and bending. Severe shearing loads and accumulation of microstrains in the crystalline lattice under compression loads led to the particles’ hardening and embrittlement, and that, in turn, resulted in their disintegration via shearing off or fracturing. The resulting smaller flattened particles were ‘cold welded’ to each other by high energy impacts from milling balls and formed ‘flake-like’ lamellar structures. Prolonged ball milling led to an increased accumulation of crystal microstrains and promoted work hardening of the particles with their embrittlement and fracturing into smaller pieces. These pieces again could be agglomerated into equiaxed solid shapes, forming ‘cabbage-like’ structures. These agglomerates were compacted by the balls’ impacts and, upon further accumulation of microstrains, could be cracked into smaller pieces. The eventual shape and size of the ball-milled metal particles were determined by the equilibrium between these competing processes—cold welding (size enlargement) and fracturing (size reduction) [83,88,112].

For the samples ball milled with the additive (10, 5, and 2.5 wt.% Rose alloy) the images were taken in the BSE mode that allowed highlighting the presence of the activator composed of heavy metals. In the said microphotographs, light gray and white colors depicted the heavy activator metals, Bi, Sn, and Pb, while the darker shades corresponded to the base scrap components (Mg and Al). The lighter regions, however, could potentially contain not only the Rose alloy traces but also sites enriched with heavy alloying elements (Nd, Zr, Zn) or those contaminated with Fe—major component of steel pieces chipped-off from the milling balls during activation. The maximum Fe contamination estimated from the changes in the balls’ masses was ~0.2 wt.%. Initially, Rose alloy presented in the form of pieces nearly several mm in size. Taking into account its ductility and low melting point of 94 °C, its spreading over particles was presumably caused by its melting by the heat released during ball milling rather than mechanical disintegration. The distribution of the alloy over the particles was determined to be non-uniform, which was not surprising for the relatively short milling durations tested in the present study. For shorter milling intervals, however, the particles’ surface regions enriched with heavy elements were generally larger in area and smaller in number, while for the maximum tested milling time they mostly represented a greater number of smaller spots.

**Figure A1:**
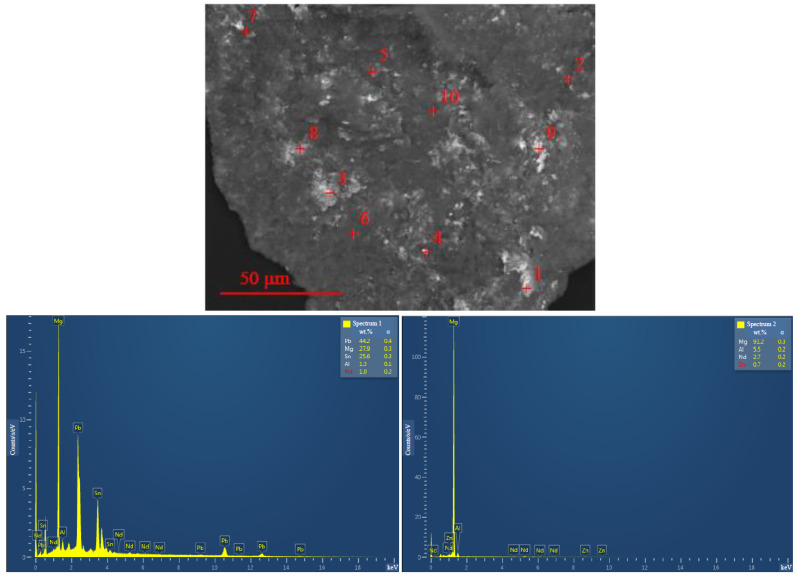

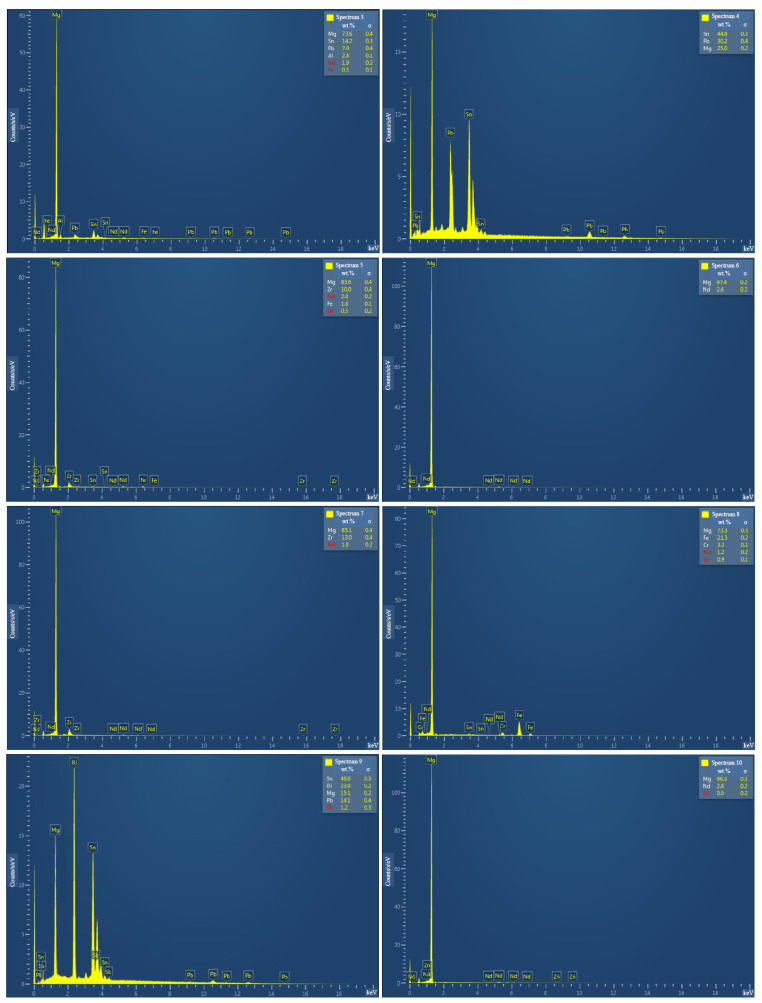
EDX analysis results (spectra) for the 1 h ball-milled sample with 10 wt.% Rose alloy.

The sample ball milled for 1 h with 10 wt.% Rose alloy was investigated by the EDX method. The results (spectra) for the selected analyzed spots are illustrated in Figure A1 (see Appendix A). The analysis revealed the presence of the major elements of scrap alloys (Mg, Al, Nd, Zr, and Zn), components of Rose alloy (Bi, Sn, and Pb), and an amount of impurities: Fe and Cr resulted from the contamination with chipped-off pieces of steel balls during ball milling, and Sb—a common impurity of Rose alloy.

#### 3.1.2. Results of XRD Analysis

**Figure 3:**
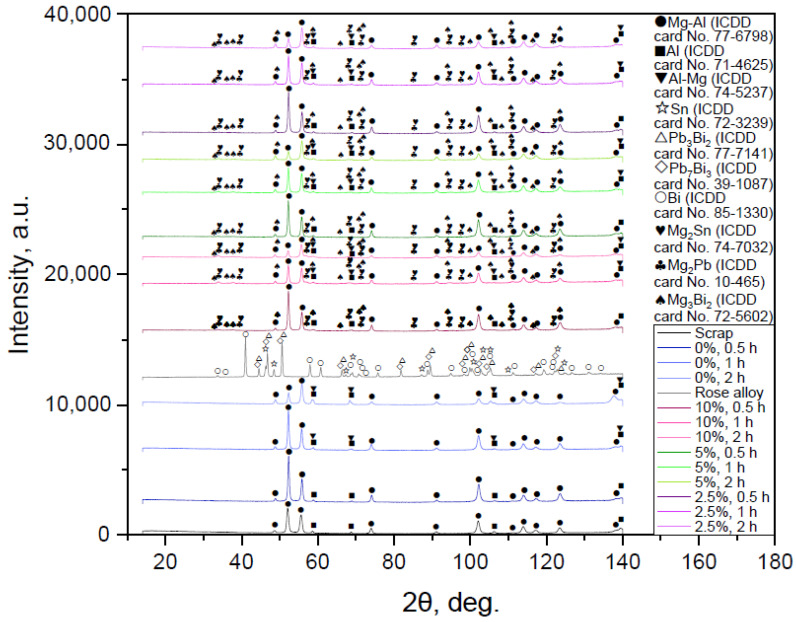
XRD patterns of the original materials, scrap and Rose alloy, and activated samples with different additive concentrations and ball-milling durations.

The XRD patterns for the samples ball milled with no additives and those containing 10, 5, and 2.5 wt.% Rose alloy are shown in Figure 3. The base phase of the original scrap and all milled samples was a solid solution of Al in the primary hexagonal close-packed Mg lattice. Peaks of the face-centered cubic Al structure were detected as well. In the cases of scrap, 0.5 and 1 h milled samples, some of the peaks for the Mg–Al and Al phases had higher intensities as compared to those for the 2 h activated sample, whose relative peak intensities were in good agreement with those from the ICDD card patterns for powders. Such a difference could arise from the partial texturization (preferred crystal orientation along a certain crystallographic direction) of the scrap’s structure, which could be ascribed to mechanical machining. Mg–Al–Zn alloys were proved to contain a solid solution of Al and Zn in Mg and precipitates of the phases Φ-Mg_21_(Al, Zn)_17_ and Mg_17_Al_12_, wherein some of the Al atoms could be replaced by Zn with the formation of Mg_17_(Al, Zn)_12_ [113,114]. Corrosion resistance of Mg–Al–Zn alloys was ascribed to the existence of intermetallic phase precipitates ‘containing’ the grains (while in small amounts they functioned as microgalvanic cells) and to protective aluminum-rich subsurface regions [115,116]. Therefore, the Al presence could probably be explained by the existence of the local Al-rich regions. For the 1 and 2 h milled samples a new phase (Mg solution in Al) appeared. The peaks’ broadening and position shift were detected as well. Such changes indicated that high energy ball milling introduced lattice microstrains and crystalline imperfections, typically favorable for pitting corrosion, into the microstructure [88,117,118]. Because of the reduction in the grain size caused by ball milling, the number (and total length) of grain boundaries increased, potentially making the material more susceptible to intergranular corrosion [119].

In Rose alloy body-centered tetragonal Sn and rhombohedral Bi crystals were definitely identified, while for the third hexagonal phase its peaks fell within the angles of two intermetallides—Pb_3_Bi_2_ and Pb_7_Bi_3_. According to the findings from [120], Pb_2_Bi was formed in the Bi–Pb–Sn system with the similar composition. Another BiPb_3_ intermetallic phase was claimed in [121]. In another study [122], the formation of BiSn (minor amount) and Pb_7_Bi_3_ was reported. However, the characteristic peaks for the latter phase were notably shifted to larger angles—the same was observed in the present study. Presumably, the third phase actually represented some blended composition.

According to the XRD patterns, the samples activated with Rose alloy demonstrated the presence of the new phases—Mg_3_Bi_2_, Mg_2_Sn, and Mg_2_Pb. The latter two intermetallides had their intensity peaks majorly overlapping. For large angles, however, the actual peaks were located between the tabulated values for those phases. The formation of the new compounds (and the absence of the original alloy phases in the samples) supported the version that the Rose alloy melted during ball milling and its components reacted with Mg. The existence of Mg_2_Pb, Mg_2_Sn, and Mg_3_Bi_2_ was confirmed for a number of Mg-based alloys [123,124,125]. The samples ball milled with smaller amounts of additive differed from those with larger ones in that their peaks for the new phases were lower.

### 3.2. Tests for Hydrogen Generation Performance

#### 3.2.1. Impact of Ball-Milling Duration

**Figure 4:**
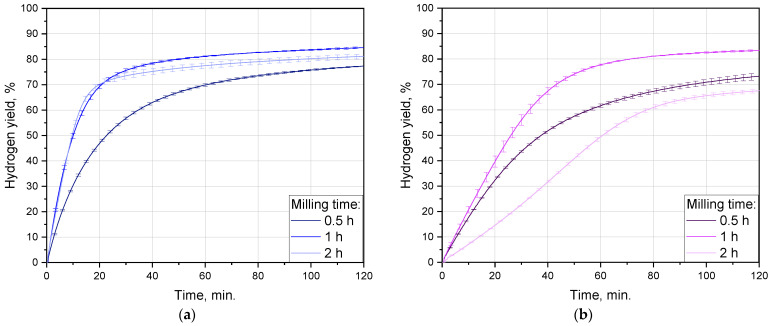

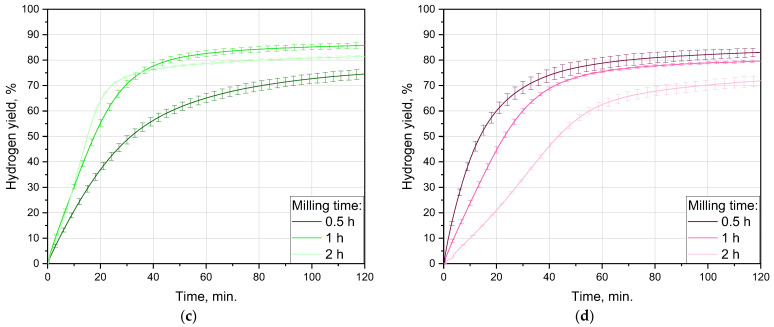
Hydrogen evolution kinetic curves for different ball-milling durations and sample compositions: (**a**) no additive; (**b**) 2.5 wt.% additive; (**c**) 5 wt.% additive; (**d**) 10 wt.% additive.

The impact of the ball-milling time interval was investigated for all Rose alloy contents: 0, 2.5, 5 and 10 wt.%. The sets of hydrogen-release kinetic curves are represented in Figure 4; the data on hydrogen generation performance are listed in Table 2. For the samples with the alloy contents of 0, 2.5, and 5 wt.% the highest maximum hydrogen evolution rates were obtained in case of 1 h activation: 86, 35, and 49 mL/min./g, respectively, while for that with the maximum 10 wt.% the value of 39 mL/min./g was the second highest. After 2 h of activation, for all the compositions those values decreased correspondingly to 77, 19, 43, and 27 mL/min./g. Milling during 0.5 h resulted in the relevant rates of 56, 31, 35, and 66 mL/min./g. After 2 h of the experiment, the achieved hydrogen yields for the samples fell within the range ~65–90%. For the samples containing 0, 2.5, and 5 wt.% Rose alloy the highest scrap-to-hydrogen conversion values also corresponded to 1 h activation time and achieved (84.6 ± 0.4)%, (83.4 ± 0.3)%, (85.8 ± 1.1)%, while again the 10 wt.% (79.6 ± 0.5)% was the second highest value. Ball milling during 2 h resulted in the relevant figures of (81.2 ± 0.9)%, (67.5 ± 0.7)%, (81.4 ± 0.5)%, and (71.9 ± 2.1)%. After 0.5 h of milling, as much as (77.3 ± 0.2)%, (73.2 ± 1.3)%, (74.5 ± 1.9)%, and (83.0 ± 1.6)% were obtained, respectively.

It was notable that for all compositions the kinetic curves for 2 h of milling had a ‘canonical’ ‘S’-like shape with a clear acceleration part in the beginning, while those for 1 and 0.5 h continuously decelerated. The mentioned curve shape was known to be typical for topochemical reactions [126]. That acceleration section could arise from the partial oxidation of the samples with residual oxygen during prolonged ball milling, because the resulting MgO took some time to be hydrated in order to form a less dense Mg(OH)_2_ [127].

On the one hand, longer milling time favored the accumulation of a greater number of crystal lattice imperfections (grain boundaries, dislocations, stacking faults, wherein the energy of the atoms is higher) vulnerable to localized corrosion attacks, e.g., pitting corrosion by the enrichment in chloride [88,118,128,129]. On the other hand, however, it was capable of enhancing partial oxidation of the powdered samples, thus reducing their reactivity. It obviously affected the distribution of the activating additive as well. In study [130], in the case of Mg–Ni powders it was assumed that, after a shorter milling, a larger cathode (Ni) surface area contributed to faster hydrogen release. However, for aluminum-based powders Bi nanoparticles were reported to demonstrate better performance than bigger, micron-sized particles [131]. In [47], an intermediate-sized copper powder occurred to provide better aluminum activation than the finer and coarser ones. Most likely, different sorts of additives might have various ‘optimal cathodic structures’ (sizes of the sites enriched with additive components and their distribution over particles) prone to inducing intensive galvanic corrosion of the base metal.

The existence of the optimal ball-milling duration was established in numerous previous studies [88,109,130]. As seen for the tested alloy contents and milling conditions, 1 h was optimal for the samples with 0, 2.5, and 5 wt.% Rose alloy since that duration provided the best hydrogen evolution performance (yields and maximum reaction rates), while in the case of the maximum 10 wt.% that optimum shifted to 0.5 h.

#### 3.2.2. Influence of Additive Content

**Figure 5:**
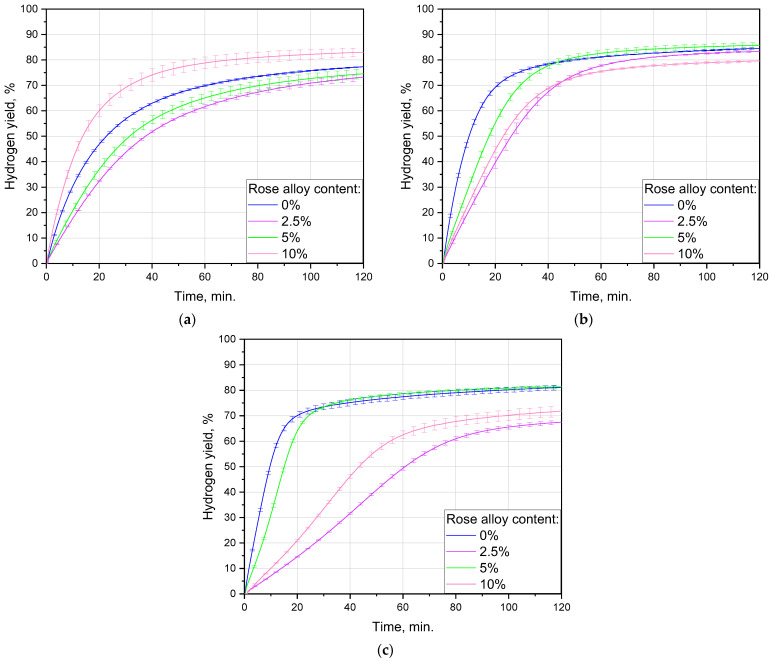
Hydrogen generation kinetic curves for different sample compositions and ball-milling time intervals: (**a**) 0.5 h; (**b**) 1 h; (**c**) 2 h.

The influence of the additive content for various milling durations is demonstrated in Figure 5. It was noteworthy that for all tested activation intervals, the lowest maximum hydrogen reaction rates were observed for the sample with 2.5 wt.% Rose alloy. At longer time intervals of 1 and 2 h, the second-lowest values corresponded to the 10 wt.% of the additive, while the highest rates were achieved for the sample without additives. For the shortest tested period of 0.5 h the fastest hydrogen evolution along with its maximum yield were obtained for the sample with 10 wt.% Rose alloy, while that without additives moved to the ‘second-best position in the rating’. The largest divergence in the hydrogen yields emerged for the samples with different additive contents after prolonged 2 h activation. It appeared to be smaller in the case of 0.5 h and was minimal for 1 h of ball milling.

Intensified oxidation of Mg and Al powders ‘mechanically coupled’ with metal additives (Ni, Co, Bi) by air at elevated temperatures was proved by thermogravimetric analysis [132,133]. Accordingly, after 2 h of milling, the highest activator amounts of 5 and 10 wt.% tested in the present study were expected to enhance oxidation of the samples with residual oxygen, and their maximum reaction rates were anticipated to decline below those for 0 and 2.5 wt.% Rose alloy contents. That forecast, however, failed to turn into reality. Therefore, to elaborate an explanation for the observed dependency of the samples’ reactivity on the Rose alloy content, other factors were considered below.

The samples with 2.5, 5, and 10 wt.% of the additive contained 0.45, 0.9, and 1.8 wt.% Sn; 0.8, 1.6, and 3.2 wt.% Pb; and 1.25, 2.5, and 5 wt.% Bi, respectively. Various amounts of those alloying elements were determined to improve or reduce the corrosion stability of various Mg-based alloys. Thus, a small Pb content of 0.1 wt.% had a negative effect on the corrosion resistance of the AZ91 alloy [134], but in the range 0.2–1 wt.% it improved the stability of the Mg–10Al–12Si system [135]. For the tested 0.6, 1.2, and 1.8 wt.% fractions of Pb added to the Mg97–Zn1–Y2 alloy, corrosion protection decreased with the increase in the additive content [124]. Increase in the Pb concentration from 2.5 to 7.5 wt.% improved the corrosion protection for the Mg–3Al alloy, but reduced it for the Mg–9Al and Mg–6Al systems [136]. According to the findings from [137,138,139,140], the Mg–6Al and Mg–6Al–1In compounds modified with 5 wt.% Pb had enhanced anodic reaction activity. In large amounts (~30 wt.%), Pb inhibited corrosion of the Mg–9.2Al–0.8B alloys [141]. The proposed mechanism of corrosion enhancement in Mg–Al alloys by Pb was associated with the formation of a complex reaction product, wherein precipitated Pb_x_O on the alloy’s surface facilitated the precipitation of Al(OH)_3_ that could ‘peel off’ dense Mg(OH)_2_ in the form of Al(OH)_3_∙2Mg(OH)_2_ [137,140].

The Mg–1Sn alloy was reported to suffer from more rapid corrosion than pure Mg, and 1–3 wt.% of Sn significantly accelerated the corrosion and hydrogen release rate of the Mg–6Al–1Zn, Mg–6Al–5Pb–0.5Mn–0.5RE, and Mg–7Al–0.2Mn compounds [142,143,144,145,146]. For the Mg–Sn alloy 1.5 wt.% Sn was determined to provide the best corrosion resistance as compared to 0.5, 1, and 2 wt.% [147]. In contrast, 3 wt.% Sn added to the Mg–2Al–6Zn and Mg–Zn alloys improved their corrosion protection, and an extruded Mg alloy modified with 5 wt% Sn demonstrated improved corrosion resistance [148,149,150]. Large Sn amounts of 5 and 10 wt.% introduced into high pure Mg were reported to improve its corrosion resistance drastically [151]. Nevertheless, in Mg-based alloys with 2–8 wt.% Sn, as the area fraction of the grain boundaries increased (i.e., grain size decreased), the amount of Mg_2_Sn particles increased and accelerated the H_2_ evolution rate [152]. Meanwhile, in another study [153] it was suggested that the grain size refinement and decrease in volume fraction of the second-phase particles could improve the corrosion resistance of Mg–Ca alloys. An important Sn property was the formation of Sn-rich intermetallics at the Mg (α-phase) grain boundaries responsible for the grain refinement. The reduced free energy barrier on the Mg_2_Sn (β-phase) surface favored the nucleation of the β-phase at the locations of the Mg_2_Sn particles. Because of that, a larger number of nucleation cores were created, thus forming a refined and dispersed β-phase hampering the grain growth [154].

In case of the AZ91 modified with Ca and Bi (1 wt.%), no corrosion was observed in the Mg_2_Ca phase while Mg_3_Bi_2_ sites were exposed to severe attack [155]. The introduction of 0.5 wt.% Bi into the Mg–1.2Ca system was reported to increase its protection against corrosion, while the larger tested values of 1.5, 3, 5, and 12 wt.% diminished it; for the concentrations of 3 wt.% and higher, a significant reduction in the grain size and enhanced dynamic recrystallization of the matrix were established as well [123,156,157]. In study [158], for the tested Mg–Bi alloys the self-discharge rates associated with hydrogen evolution were the lowest for 0.5 wt.% and gradually increased for 1 and 2 wt.%. Minor amounts of Bi, 0.2–1 wt.%, added to the Mg–8Al system demonstrated that the optimal additive content was 0.4 wt.% [159], and in pure Mg 0.8 wt.% Bi provided excellent anticorrosion properties [160]. In study [97] it was concluded that for as-cast and as-rolled Mg–6Bi–2Sn alloys their respective predominant corrosion modes were intergranular and pitting corrosions. Another finding was that the refined grain structure and finely dispersed secondary phase particles with considerably smaller sizes and lower volume fraction enhanced the corrosion resistance of the as-rolled alloy. The formation of complex passive films (MgO, Mg(OH)_2_, SnO_2_, and Bi_2_O_3_) contributing to corrosion stability was assumed in [97,160]. It was established as well that Bi addition yielded significant strength improvement to Mg alloys produced via extrusion (plastic deformation at elevated temperature took place during ball milling as well). At the same time, large amounts of undissolved coarse Mg_3_Bi_2_ particles reduced their ductility because they acted as cracking sources [161,162].

According to the findings from [163], low (0.1–0.5 wt.%) amounts of Pb, Sn, or Bi introduced into a pure (99.95%) Mg alloy augmented its corrosion protection. It was noteworthy that in the BSE microphotographs of the alloys with 0.5 wt.% additive, no irregularities for the samples with Sn and Pb were observed, while they were clearly visible in case of Bi. Such a discrepancy could be ascribed to the high solubility of Pb and Sn in the Mg lattice as compared to that for Bi (maximum 7.75, 3.35, and 1.12 wt.%, respectively).

Summarizing the abovementioned data, one could come to a conclusion that for different alloy systems, within some content ranges the additives had a positive influence on the corrosion resistance, while other amounts had the opposite effect. The combined impact of grain size and secondary phase properties on the corrosion behavior of Mg-based alloys generally remained unclear. As to the grain-refining properties of Sn and Bi, it was difficult to derive whether they introduced any contributions to the grain refining provided by severe plastic deformations during high energy ball milling or not. Moreover, their effects were considered for solidification of alloys produced by melting and not for ‘mechanical alloying’ via ball milling.

The lowest hydrogen generation performance in the case of the minimum 2.5 wt.% Rose alloy could be associated with the small Sn and Pb amounts that fell within the ranges favorable for corrosion stability, while the Bi content was close to the ‘borderline’ between ‘positive’ and ‘negative’ impacts. According to the data from [164], Pb solubility in Mg at 100 °C was as high as 0.4 at.%, i.e., 3.3 wt.%, which was quite a high value. At room temperature it apparently was lower; however, its large solubility probably resulted in the formation of a negligible Mg_2_Pb amount in the sample with 2.5 wt.% (and 5 wt.%) Rose alloy, if any at all. As the XRD patterns for Mg_2_Pb and Mg_2_Sn overlapped, those phases could not be distinguished. Therefore, for the samples with relatively low Rose alloy contents, Pb possibly did not contribute to galvanic corrosion by the formation of Mg_2_Pb. The effect of Pb atoms dissolved in the Mg lattice on its corrosion behavior still was not clearly explained; however, it might be similar to that proposed for Sn. The solubility of Sn at room temperature was ~0.17 wt.% [165]. In study [154], it was stated that Sn in the form of Sn_2_Mg (~0.2 wt.% of total 0.5 wt.%) could promote galvanic corrosion of Mg, while Sn in a solid solution imposed an opposite effect (stabilization of the Mg matrix with incorporated Sn atoms also still lacks a detailed explanation). So, it could turn out that considerable amounts of Sn and Pb merely dissolved in the Mg lattice (indeed, the respective XRD peaks for Sn_2_Mg and Mg_2_Pb were barely visible), and generally their impact on the corrosion promotion was either neutral or negative. Considering a low solubility of 0.1 wt.% at room temperature [166] and visible respective diffraction peaks, Bi definitely formed a Mg_3_Bi_2_ phase. In the case of that phase, its influence on the Mg corrosion intensification could extend from neutral to negative because of the following reasons. As it was established in the studies discussed above, small-sized and evenly distributed Mg_3_Bi_2_ particles could be beneficial for corrosion protection. First, in contrast with the coarse particles, they did not represent cracking sources promoting corrosion. Second, their ability to form more uniform and continuous complex passive films of MgO, Mg(OH)_2_, and Bi_2_O_3_ (by the oxidation of the samples’ components with residual air during ball milling) was higher. Third, if the protection film was destroyed, although Mg_3_Bi_2_ particles provided sites for the pitting corrosion initiation, they represented too small ‘cathodic sites’, thus hampering the corrosion pits’ growth, and those pits could be ‘shielded’ by the mentioned components of the passive films. In the case of the minimum Rose alloy content, the additive was majorly distributed over the particles in the form of small sites presumably resistant to severe corrosion attacks.

For the samples with the maximum 10 wt.% additive, their poor efficiency after 1 and 2 h could have the following possible explanation. The longer milling durations resulted in more uniform distribution of the secondary phase regions with considerably smaller sizes over the particles. As mentioned above, even distribution of fine Mg_3_Bi_2_ particles was beneficial for the formation of more uniform and continuous complex passive films of MgO, Mg(OH)_2_, and Bi_2_O_3_, thus providing better corrosion resistance. For the samples under discussion, because of the larger amount, Sn could contribute as well by forming another film’s component, SnO_2_. Longer activation intervals resulted in significant particles’ ‘reshaping’ from flattened shapes into equiaxed compacted solid objects that reduced their specific surface area. A hypothetical effect for the high activation content could be local cracking of the material in the vicinity of the coarse Mg_3_Bi_2_ particles formed in the first 0.5 h of ball milling. Such embrittled regions could contribute to the mechanical embrittlement from steel ball impacts and accelerate the mentioned microstructural transformations owing to more intensive fracturing. This could potentially lead to a more rapid decrease in the specific surface area of the samples.

The excellent result for the sample with 10 wt.% Rose alloy and 0.5 h activation could be ascribed to the following reasons. First, it had a relatively large specific surface area because the originally flat particles did not pass through drastic transformations after the relatively short milling time of 0.5 h (proved by the SEM analysis). Second, the said embrittlement in the vicinity of the Mg_3_Bi_2_ phase (which was likely deposited in the form of coarse particles because of the additive abundance and uneven distribution after a short milling time) could result in the formation of crevices, thus enlarging the area in contact with the aqueous solution (hypothesis). Third, the complex passive films (MgO, Mg(OH)_2_, SnO_2_, and Bi_2_O_3_) determined to improve corrosion resistance were not formed in a continuous and/or uniform manner after such a short milling time (hypothesis). Fourth, the formed large-sized regions enriched with the intermetallic phases Mg_3_Bi_2_, Mg_2_Sn, and Mg_2_Pb could be beneficial for intensive corrosion with hydrogen release (hypothesis). As the optimal sizes of ‘cathodic’ sites were determined to be the smallest (Al milled with Bi) [131], the largest (Mg–Ni system) [130], and intermediate (Al activated with Cu) [47] in different relevant studies on hydrogen generation, further studies are needed to support or abandon the idea of the optimal sizes and distributions of the ‘cathodic’ sites.

As to the samples with the intermediate 5 wt.% Rose alloy content ball milled for 1 and 2 h and their lower performance as compared to those without additives, the potential reason again could be the formation of the abovementioned complex passive oxide films. However, because of the lower concentrations of the additive components, those films were possibly removed more readily as compared to those for the compounds with 10 wt.% additive. Presumably, this provided the acceleration of the reaction rates for these samples and caused their kinetic curves to match (within error bars) those for the samples without additives along their deceleration sections. The underperformance of the 0.5 h milled sample was quite a complex question. Based on the above data, it could be assumed that some other effects determined by the concentrations of the separate Rose alloy components, Pb, Sn, and Bi, on the Mg alloys corrosion behavior could come into the spotlight. The findings from the different studies discussed above did not provide a complete picture of the dependency of the corrosion resistance or intensification on the contents of the listed metals. It could be that in the lack of mechanical activation (as compared with longer milling durations), the corrosion behavior of that sample was governed by a combination of some protective and destructive effects imposed by partially dissolved Pb and Sn (found to have enhanced corrosion resistance), minor Mg_2_Sn and, probably, Mg_2_Pb phases, and the predominant Mg_3_Bi_2_ compound, which could produce quite controversial effects. Therefore, for clear understanding of the corrosion behavior of Mg-based alloys affected by Sn, Pb, and Bi, future investigations are needed.

To provide further insights into the reasons for the observed non-monotonic hydrogen production dependency on the additive concentration along with ball milling, some additional techniques can be employed. One of them could be X-ray photoelectron spectroscopy (XPS) for the investigation of the complex oxide films that could be formed on the particles’ surfaces. Another useful procedure could be mixing the particles with carbon and resin, polishing the solidified mixture, and investigating the particles’ cross-sections by a metallographic microscope to clarify the distribution of the intermetallic phases. Since only obviously large deviations in the particles’ sizes could be detected by visual inspection through a microscope, accurate size measurements for large quantities of particles of different samples could also provide valuable information on whether the larger additive content accelerated microstructural transformation during ball milling or not.

### 3.3. Characteristics of the Reaction Products

**Figure 6:**
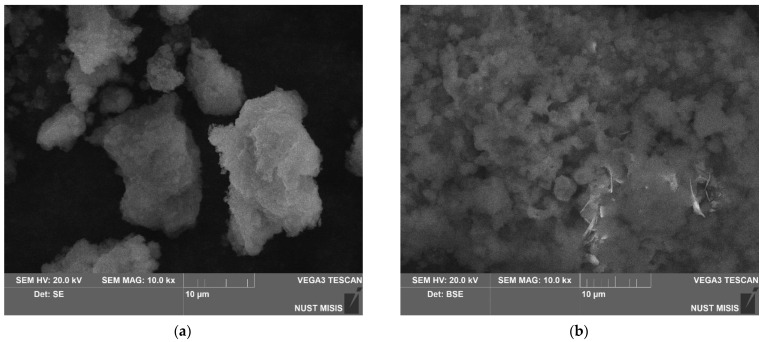
Microstructure of the solid reaction product obtained from different samples: (**a**) no additives (SE); (**b**) 5 wt.% Rose alloy (BSE).

The microphotographs of the reaction products from the 1 h milled samples without additives and with 5 wt.% Rose alloy are shown in Figure 6. The product was deposited in the form of agglomerates of small ‘flakes’. In the BSE image for the sample with Rose alloy, the solid product composed of the base metal, hydrogen, and oxygen (shown in gray shades) contained a structure of large ‘lamellas’ (shown in white). These ‘lamellas’ likely were Mg compounds with heavier elements (Mg_3_Bi_2_, Mg_2_Sn, and Mg_2_Pb), as their contrasting light color indicated the presence of heavy chemical elements.

**Figure 7:**
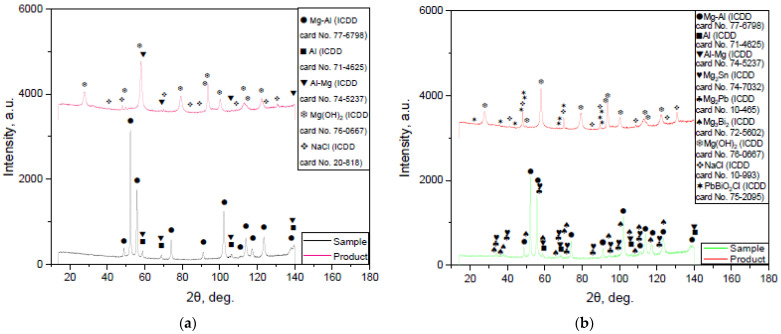
XRD patterns of the solid reaction product obtained from different samples: (**a**) no additives; (**b**) 5 wt.% Rose alloy.

The XRD patterns for the products are given in Figure 7. The product majorly comprised Mg(OH)_2_, as expected. Residual NaCl from the solution was identified as well. None of the intermetallic phases was detected. However, their presence was proved by the microphotograph. Presumably, their amounts in the samples appeared to be too low owing to their possible ‘exfoliation’ in the course of the reaction. In [159], the formation of BiOCl for Mg-based materials reacting with 3.5 wt.% NaCl solution was revealed. In the present study, a third compound matching the XRD pattern for the product obtained from the sample with Rose alloy was PbBiO_2_Cl.

## 4. Conclusions

Twelve types of samples for hydrogen generation were elaborated of the mixed scrap of Mg–Al–Zn and Mg–Nd–Zr–Zn casting alloys with 0, 2.5, 5, and 10 wt.% low melting point Rose alloy by high energy ball milling during 0.5, 1, and 2 h. Their reactivity in 3.5 wt.% aqueous NaCl solution was tested at room temperature. The impacts of additive content and milling time were investigated.

The main influence from ball milling was the creation of imperfections in the crystal lattice of the base metal that increased its vulnerability to pitting corrosion, structural evolution of the particles from flat shapes to compacted solid objects that diminished their specific surface area, and partial surficial oxidation of the particles with residual oxygen during ball milling that decreased the reaction rates in the beginning. For all samples except that with 10 wt.% Rose alloy, 1 h of activation provided the highest hydrogen yields and reaction rates. For the sample with the maximum additive content, 0.5 h appeared to be optimal as it provided the maximum hydrogen yield and rapid reaction progress.

The impact of the additive content was complex. The lowest amount of 2.5 wt.% Rose alloy produced a negative effect on the hydrogen release of the samples for all ball-milling durations. The reason for this could be the considerable dissolution of Pb and Sn in the Mg crystal lattice with the formation of a solid solution inhibiting corrosion, together with the formation of fine sparsely distributed Mg_3_Bi_2_ particles generally resistant to severe corrosion attacks. The overperforming result for the sample with the maximum 10 wt.% additive content and 0.5 h activation could be ascribed to the following reasons. First, it had a relatively large specific surface area because no drastic transformations took place after the relatively short milling time. Second, embrittlement in the vicinity of the Mg_3_Bi_2_ phase (likely deposited as coarse particles owing to the additive abundance and uneven distribution after a short milling time) could result in the formation of crevices, thus enlarging the area in contact with the aqueous solution. Third, the short milling time could be insufficient for the formation of the complex passive films (MgO, Mg(OH)_2_, SnO_2_, and Bi_2_O_3_). Fourth, the observed large-sized regions enriched with the intermetallic phases Mg_3_Bi_2_, Mg_2_Sn, and Mg_2_Pb could turn out to be beneficial for the intensive corrosion with hydrogen release. The underperformance of the samples with 10 wt.% Rose alloy after 1 and 2 h milling was associated with the following effects. First, structural particle transformations during prolonged ball milling involved a decrease in their specific surface area (this process might be promoted by the embrittlement coming from the cracking sources of the coarse Mg_3_Bi_2_ particles). Second, prolonged ball milling could result in a much more uniform distribution of the small-sized intermetallic phases Mg_3_Bi_2_, Mg_2_Sn, which contributed to the formation of a dense and continuous complex oxide film (MgO, Mg(OH)_2_, SnO_2_, and Bi_2_O_3_). As to the samples with the intermediate 5 wt.% additive content milled for 1 and 2 h and their underperforming the samples without additives, the potential reason again could be the formation of the abovementioned complex passive oxide films. No reliable explanation for the 0.5 h milled sample was elaborated. It could be that in the lack of mechanical activation, the corrosion behavior of that sample was governed by a combination of some protective and destructive effects imposed by partially dissolved Pb and Sn (found to have enhanced corrosion resistance), minor Mg_2_Sn and, probably, Mg_2_Pb compounds, and the predominating Mg_3_Bi_2_ phase, which could produce quite controversial effects. Therefore, to provide a clear understanding of the corrosion behavior of Mg-based alloys affected by Sn, Pb, and Bi, further studies should be carried out.

The findings of the present study could be interesting as another contribution to the existing knowledge about Mg-based material corrosion behavior. It was shown that intermetallic structures that were expected to enhance Mg corrosion with hydrogen release could impose quite the opposite effect. Potentially, the corrosion inhibition properties of Rose alloy can be studied for Mg-based materials manufactured by some plastic deformation techniques (e.g., friction stir processing, cold or hot rolling). The major challenge could be associated with the elaboration of such materials not via a laboratory ball mill but using a large industrial ball mill. Variation in milling conditions can significantly affect the properties of the resulting composite materials. The findings of the present study, however, can be potentially extended or applied to other types of composite or waste materials. For example, the waste of some other Mg alloy grades can be activated in a similar manner, and some close compositions of the activating alloy can be used. Summarizing the above, the investigated method can be employed for the utilization of mixed Mg-based waste with hydrogen generation.

## Figures and Tables

**Table 1:** Parameters of experiments, ball milling, and samples.

Milling Speed, rpm	Balls Diameter, mm	Balls-to-Powder Mass ratio	Powder Portion Mass, g	Milling Time, h	Rose Alloy Content, wt.%	Experiment Temperature, °C	Aqueous Solution	Sample Mass, g
580	10	24:1	4	0.5; 1; 2	0; 2.5; 5; 10	25	3.5 wt.% NaCl	0.75

**Table 2:** Hydrogen generation performance of the samples with different compositions and milling durations.

Composition	Ball-Milling Time, h	Hydrogen Yield, %	Maximum Reaction Rate, mL/g/min.
Scrap without additives	0.5	77.3 ± 0.2	56
	1	84.6 ± 0.4	86
	2	81.2 ± 0.9	77
Scrap + 2.5 wt.% Rose alloy	0.5	73.2 ± 1.3	31
	1	83.4 ± 0.3	35
	2	67.5 ± 0.7	19
Scrap + 5 wt.% Rose alloy	0.5	74.5 ± 1.9	35
	1	85.8 ± 1.1	49
	2	81.4 ± 0.5	43
Scrap + 10 wt.% Rose alloy	0.5	83.0 ± 1.6	66
	1	79.6 ± 0.5	39
	2	71.9 ± 2.1	27

## Data Availability

Not applicable.

## Appendix A

EDX analysis results for the selected spots on the samples ball milled with 10 wt.% Rose alloy during 1 h are shown in Figure A1.
